# Proposed fibrinogen standard material with purified fibrinogen for plasma fibrinogen measurement on coagulation analyser

**DOI:** 10.1155/S146392460300018X

**Published:** 2003

**Authors:** Masahiro Okuda, Yahiro Uemura, Noriyuki Tatsumi, Susumu Honda

**Affiliations:** 1 Product Development Sysmex Corporation 1-1-2 Murotani Nishi-ku Kobe 651-2241 Japan; 2 Research and Development Division International Reagents Corporation 1-1-2 Murotani Nishi-ku Kobe 651-2241 Japan; 3 Department of Clinical and Laboratory Medicine Osaka City University Medical School Asahi-machi Abeno-ku Osaka 545-8585 Japan; 4 Faculty of Pharmaceutical Science Kinki University 3-4-1 Kowakae Higashi-Osaka Osaka 577-8502 Japan

## Abstract

A commercially available fibrinogen standard calibrated by a World Health Organization (WHO) reference material is widely used in Japan, and most clinical laboratories use the Clauss method for plasma fibrinogen measurement. However, a current issue in fibrinogen measurement is poor laboratory-to-laboratory variability. To improve the reliability of fibrinogen values and thereby solve the poor precision and accuracy of plasma fibrinogen testing, the present paper develops a simple and large preparation procedure for a suitable fibrinogen standard and quality control material and evaluates their basic performance. With a new procedure getting high purified fibrinogen by glycine precipitation, the calibrator determined by both the Clauss and Jacobson methods produced a fibrinogen concentration of 2.20 g l^−1^. The total precision of the calibrator was excellent (coefficient of variation 1.4-2.1%) in comparison with current plasma fibrinogen materials from the WHO (#98/612) and with a commercial standard (CV 1.9-3.9%). The within-run precision of the calibrator on the coagulation analysers was 1.7-2.8%. Within-analyser variability among the five instruments had good consistency (mean 2.20 ± 0.022 g l^−1^; CV 1.0%). The degradation study of the calibrator suggested that storage at 9^°^C for two years was as predicted. In conclusion, the results show that the calibrator prepared herein can be useful as a candidate Japanese fibrinogen standard and is applicable to automated and semi-automated coagulation analysers. Additionally, it is expected that it will be widely used in Japan by diagnostic manufacturers and clinical laboratories as a recommended secondary standard to estimate a fibrinogen value according to the WHO primary standard.


Journal of Automated Methods & Management in Chemistry
Vol. 25, No. 5 (September–October 2003) pp. 103–107

### Proposed fibrinogen standard material with purified fibrinogen for plasma fibrinogen measurement on coagulation analyser

**Masahiro Okuda[1,2], Yahiro Uemura[2], Noriyuki Tatsumi[3] and Susumu Honda[4]**

[1]*Product Development, Sysmex Corporation, 1-1-2 Murotani, Nishi-ku, Kobe 651-2241, Japan*
[2]*Research and Development Division, International Reagents Corporation, 1-1-2 Murotani, Nishi-ku, Kobe 651-2241, Japan*
[3]*Department of Clinical and Laboratory Medicine, Osaka City University Medical School, Asahi-machi, Abeno-ku, Osaka 545-8585, Japan*
[4]*Faculty of Pharmaceutical Science, Kinki University, 3-4-1 Kowakae, Higashi-Osaka, Osaka 577-8502, Japan*

*A commercially available fibrinogen standard calibrated by a World Health Organization (WHO) reference material is widely used in Japan, and most clinical laboratories use the Clauss method for plasma fibrinogen measurement. However, a current issue in fibrinogen measurement is poor laboratory-to-laboratory variability. To improve the reliability of fibrinogen values and thereby solve the poor precision and accuracy of plasma fibrinogen testing, the present paper develops a simple and large preparation procedure for a suitable fibrinogen standard and quality control material and evaluates their basic performance. With a new procedure getting high purified fibrinogen by glycine precipitation, the calibrator determined by both the Clauss and Jacobson methods produced a fibrinogen concentration of 2.20 g $l^{-1}$. The total precision of the calibrator was excellent (coefficient of variation 1.4–2.1%) in comparison with current plasma fibrinogen materials from the WHO (#98/612) and with a commercial standard (CV 1.9–3.9%). The within-run precision of the calibrator on the coagulation analysers was 1.7–2.8%. Within-analyser variability among the five instruments had good consistency (mean $2.20 \pm 0.022$ g $l^{-1}$; CV 1.0%). The degradation study of the calibrator suggested that storage at 9°C for two years was as predicted. In conclusion, the results show that the calibrator prepared herein can be useful as a candidate Japanese fibrinogen standard and is applicable to automated and semi-automated coagulation analysers. Additionally, it is expected that it will be widely used in Japan by diagnostic manufacturers and clinical laboratories as a recommended secondary standard to estimate a fibrinogen value according to the WHO primary standard.*

### Introduction

Fibrinogen is a heterogeneous glycoprotein of molecular weight 280 000–340 000 with three pairs of polypeptide chains (A$\alpha$, B$\beta$, $\gamma$) [1]. Plasma fibrinogen measurement is a popular coagulation test and many laboratories in Japan use Clauss's method for the measurement. In 1992, the first International Fibrinogen Standard [2] was introduced by the UK National Institute for Biological Standards and Control (NIBSC) and it is widely used in many laboratories. However, the current issue of fibrinogen measurement is the poor precision that has been reported by the Japan National External Quality Assurance Surveillance [3], and the accuracy and precision of the annual results reported by the Japanese Medical Association (JMA) surveys conducted over the past ten years are not improved [4]. Recently, the Hematology Subcommittee of the Standardization Committee of the Japan Society of Laboratory Medicine (JSLM) documented a standardized method of fibrinogen measurement in plasma and established a reliable fibrinogen standard for both the Clauss method and turbidimetric immunoassay [5,6]. Therefore, the Japanese national fibrinogen standard (calibrator) was prepared for the purpose [7] and the authors' clinical trials were reported [8,9]. The aim of the present paper was to demonstrate the commutability and precision among coagulation analysers for the calibrator of the standardization of plasma fibrinogen measurement.

* To whom correspondence should be addressed.
e-mail: m-okuda@irc-net.co.jp

### Experimental

### *Chemical reagent*

The following reagents were used: glycine, ethanol, Tween 80 and Tri-*n*-butyl phosphate (TNBP) (Kishida Chemical, Inc, Osaka, Japan), unfractionated heparin (Aventis Pharma, Tokyo, Japan), and lysine-Sepharose CL-6B (Amersham Pharmacia Biotec, Tokyo, Japan). Other reagents used were of general grade.

### *Preparation of fibrinogen standard*

A fibrinogen standard material was prepared from fresh human plasma (Alpha Therapeutic Corporation, LA, USA) according to a previous method [7]. In brief, Cohn fraction I [10] was suspended in 55 mM citrate buffer, pH 6.4, containing 100 mmol $l^{-1}$ NaCl and 12 U $ml^{-1}$ unfractionated heparin in an ice bath, and then centrifuged. The supernatant was treated by a solvent detergent of 1% (v/v) Tween 80 and 0.3% (v/v) TNBP for virus inactivation [11]. Glycine (1.8 mol $l^{-1}$) was then added to the treated solution and centrifuged. This process was repeated twice and the final precipitates collected. Precipitated fibrinogen was dissolved in 20 mmol $l^{-1}$ citrate buffer, pH 6.8, containing 100 mmol $l^{-1}$ NaCl and passed through a lysine-Sepharose column ($2.5 \times 15$ cm, 50 ml) to remove plasminogen (figure 1).

http://www.tandf.co.uk/journals
DOI: 10.1080/14639240310001641843

Fresh human plasma
↓
Cohn fraction I
↓
Virus inactivation at 30°C for 1 h
(treated with 1% (v/v) Tween 80 and 0.3% (v/v) TNBP)
↓
Glycine precipitation
(repeat three times)
↓
Removal of plasminogen
(lysine-Sepharose column)
↓
Sterile filtration
↓
Purified fibrinogen solution
↓
Lyophilization

(approximately 2.20 g $l^{-1}$)

*Figure 1. Fibrinogen purification procedure.*

The test preparation was prepared from the purified fibrinogen to a concentration of 2.20 g $l^{-1}$ in a matrix of 20 g $l^{-1}$ protease-free bovine serum albumin (Serologicals Corporation, GA, USA) in 20 mmol $l^{-1}$ citrate buffer, pH 6.8, containing 100 mmol $l^{-1}$ sodium chloride. It was then dispensed into silicone-coated glass vials, frozen and lyophilized.

*Reference material*

The materials used for calibration, both prepared from human plasma, were the World Health Organization (WHO) fibrinogen international standard (#98/612) (NIBSC, Potters Bar, UK) [12] and the commercial fibrinogen standard (Fib standard™, Sysmex Corporation, Kobe, Japan). Each vial was reconstituted carefully according to the handling instructions.

*Fibrinogen measurement*

The calibrators were diluted to five calibration concentrations (1/5, 1/10, 1/15, 1/20, 1/30 dilutions) using Owren's veronal buffer. The calibration assay of the thrombin-clottable rate originally described by Clauss [13] was conducted according to National Committee for Clinical Laboratory Standards (NCCLS) procedures [14] using a Thrombocheck Fib(L) (Sysmex) [15]. The CA-1500, CA-7000 (Sysmex), Coagrex-700 and Coagrex-800 (Shimadzu Corporation, Kyoto, Japan) [16] for a fully automated coagulation analyser, and KC-4a micro (Amelung GmbH, Lemgo, Germany) for a semi-automated coagulation analyser were used. The clottable fibrinogen concentration was determined by Jacobsson's method [17] using a UV-160 spectrophotometer (Shimadzu).

*Precision comparison between commercial materials and the test preparation*

The within-run ($n = 20$) and between-day ($n = 5$) precisions in commercial fibrinogen standard materials, the WHO standard (#98/612), the Fib standard™ and the test preparation were examined on Coagrex-800.

*Precision comparison among coagulation analysers*

The within-run precision ($n = 20$) of the test preparation was examined on five kinds of coagulation analysers (Coagrex-700, Coagrex-800, CA-1500, CA-7000, KC-4a micro) using the Clauss and Jacobsson methods.

*Degradation study*

The accelerated stability test of the test preparation was examined after 0, 1, 2, 4 and 6 months of storage at −20, 4, 20, 37 and 45°C using the Coagrex-800 automated coagulation analyser. Five vials stored at each temperature were evaluated by the Clauss method. The long-term stability was predicted using the Arrehenius equation [18].

*Statistics*

A Mann–Whitney $U$-test was used for comparison.

### Results

*Precision comparison between commercial materials and the test preparation*

The coefficient of variations (CVs) for within-run ($n = 20$) and between-day ($n = 5$) precisions of the WHO standard (#98/612), Fib standard™ and test preparation were 1.9–3.6, 2.7–3.9 and 1.4–2.1%, respectively (table 1).

Parallel calibration curves for the WHO standard (#98/612) and the test preparation are shown in figure 2.

*Precision comparison among coagulation analyses*

The CVs for within-run ($n = 20$) of the test preparation using Coagrex-700, Coagrex-800, CA-1500, CA-7000 and KC4a micro were 1.72, 2.43, 2.35, 2.71 and 2.71%, respectively (table 2), while the CV determined by the Jacobsson method was 4.37%. The statistical mean (g $l^{-1}$) between instruments showed no significant difference versus the Jacobsson method by the $U$–test. The CV of between-analyser precision (five instruments) for the test preparation was 1.00% (table 3).

*Comparison of the fibrinogen concentration measured by the Clauss and Jacobsson methods*

No significant difference of fibrinogen concentration between the Clauss and Jacobsson methods by $U$-test was observed (table 4).

*Degradation study*

Table 5 shows that the fibrinogen potency ratio of the test preparation determined by the Clauss method was indicated at various stressed temperatures relative to the reference stored at −20°C. It is suggested that the test preparation is stable for one year at 20°C and no

*Table 1. Precision between fibrinogen standard materials on Coagrex-800.*

| | Purified material | | Plasma material | | | |
|---|---|---|---|---|---|---|
| | Test preparation | | WHO (#98/612) | | Fib standard$^{TM}$ | |
| | Within-run | Between-day | Within-run | Between-day | Within-run | Between-day |
| Mean ($g\,l^{-1}$) | 2.20 | 2.21 | 2.21 | 2.20 | 2.32 | 2.31 |
| SD | 0.045 | 0.032 | 0.079 | 0.042 | 0.089 | 0.064 |
| CV(%) | 2.05 | 1.45 | 3.57 | 1.91 | 3.84 | 2.78 |

Mean of within-run and between-day reproducibility is expressed as fibrinogen concentration ($g\,l^{-1}$). The within-run was replicated 20 times and the between-day for five days.

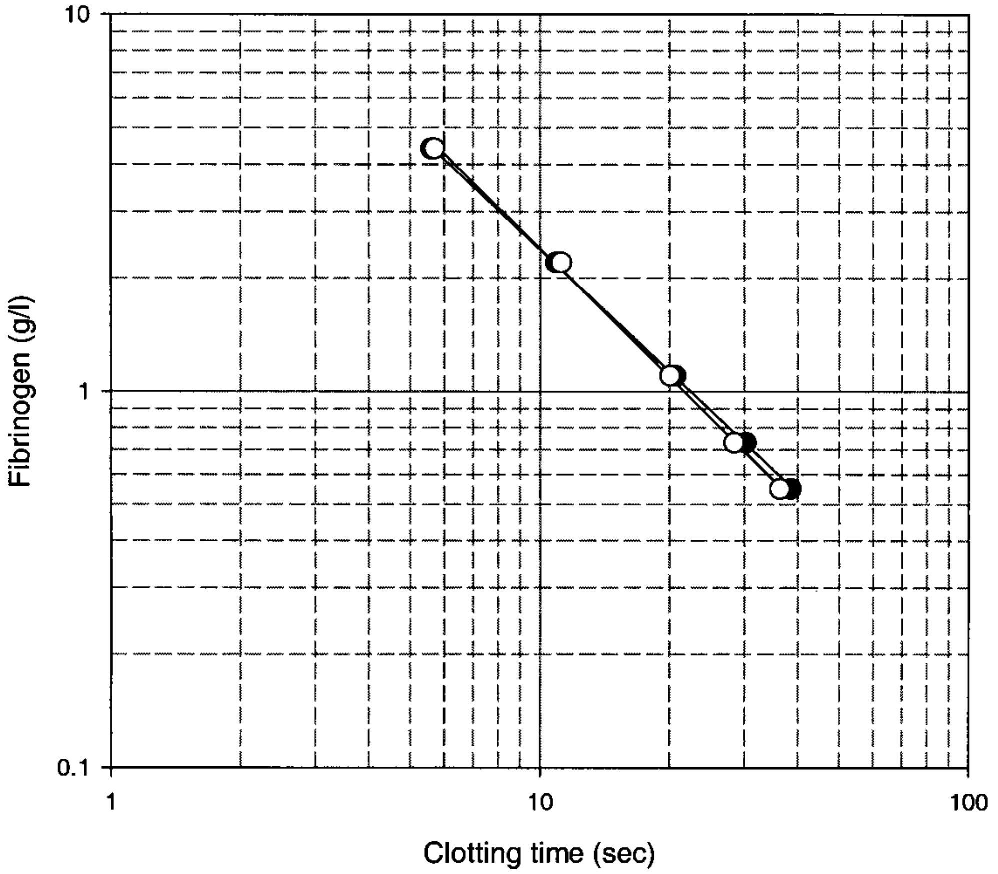

*Figure 2. Comparison of calibration curves by Clauss's method on Coagrex-800: •, WHO standard (#98/612); ○, Test preparation.*

*Table 2. Precision comparisons of the test preparation among coagulation analysers.*

| | Methods | Mean ($g\,l^{-1}$) | SD | CV (%) | Mann–Whitney *U*-test |
|---|---|---|---|---|---|
| Manual method | Jacobsson | 2.22 | 0.097 | 4.37 | * |
| Automated analyser | Coagrex-700 | 2.21 | 0.038 | 1.72 | n.s. |
| | Coagrex-800 | 2.22 | 0.054 | 2.43 | n.s. |
| | CA-1500 | 2.17 | 0.051 | 2.35 | n.s. |
| | CA-7000 | 2.18 | 0.059 | 2.71 | n.s. |
| Semi-automated analyser | KC-4a micro | 2.21 | 0.060 | 2.71 | n.s. |

n.s., No significance versus mean of the Jacobsson method (*).
Mean is expressed as fibrinogen concentration ($g\,l^{-1}$) and was replicated 20 times. Fibrinogen concentrations were calculated by WHO standard (#98/612). The Jacobsson method is a basic procedure for fibrinogen standardization based on a gravimetric method.

*Table 3. Between-analyser precisions in the test preparation.*

| $n$ | Mean ($g\,l^{-1}$) | SD | CV (%) |
|---|---|---|---|
| 5 | 2.20 | 0.022 | 1.00 |

Mean is expressed as the fibrinogen concentration ($g\,l^{-1}$). Five kinds of coagulation analysers (Coagrex-700, Coagrex-800, CA-1500, CA-7000 and KC-4a micro) were used. The results were calculated based on table 2.

*Table 4. Comparison of fibrinogen concentration of the test preparation.*

| Clauss (C) | Jacobsson (J) | C/J (%) | Mann–Whitney *U*-test |
|---|---|---|---|
| 2.20 ± 0.022 | 2.22 ± 0.097 | 99.1% | n.s. |

n.s., No significance versus Jacobsson (J).
Results of Clauss (C) and J were according to tables 2 and 3. Mean is expressed as fibrinogen concentration ($g\,l^{-1}$) and was replicated 20 times.

*Table 5. Degradation stability of the test preparation.*

| | Storage (months) | | | | |
|---|---|---|---|---|---|
| Temperature (°C) | 0 | 1 | 2 | 4 | 6 |
| −20 | **1.00** | 1.00 | 0.99 | 1.00 | 0.99 |
| 4 | 1.00 | 0.99 | 1.00 | 1.00 | 0.98 |
| 20 | 1.00 | 0.99 | 0.99 | 0.98 | 0.97 |
| 37 | 1.00 | 0.98 | 0.97 | 0.95 | 0.93 |
| 45 | 1.00 | 0.95 | 0.93 | 0.91 | 0.83 |

Means were calculated against the relative temperatures of the fibrinogen standard stored at −20°C. The bold figure indicates the reference storage.

*Table 6. Predicted storage of the test preparation.*

| Temperature (°C) | Predicted storage (years) |
|---|---|
| 4 | 3.2 |
| 9 | 2.2 |
| 11 | 1.8 |
| 20 | 1.0 |
| 30 | 0.5 |

Predicted storage was calculated according to the Arrhenius equation.

considerable loss is observed at 37°C for four months and at 45°C for one month (table 5). The test preparation approximately predicted storage at 9°C for two years (table 6).

### Discussion

Fibrinogen is a heterogeneous plasma protein with three pairs of polypeptide chains and is a crucial factor for prothrombin time and activated partial thromboplastin time assays. Many laboratories in Japan use Clauss's method for plasma fibrinogen measurement on an automated coagulation analyser. However, its determination is qualitative not qualitative. A very high variation in its surveillance results was observed. A current issue in fibrinogen measurement was the poor precision reported in various surveys [3,4]. The Haematology Subcommittee of the Standardization Committee of the JSLM has published a proposed standard method for the measurement of fibrinogen in plasma [5,6]. The present authors reported a Japanese national fibrinogen standard with purified fibrinogen for the purpose [7]. Thus, it is suggested that a suitable standard might help to improve the precision and accuracy of fibrinogen measurement in clinical laboratories [8].

After preparing the test preparation derived from purified fibrinogen, its measurement precision was compared using five kinds of coagulation analysers. The test preparation showed a parallel calibration curve with the WHO (#98/612) standard (figure 2) and superior precision to plasma materials for all within-run and between-day measurements (table 1). The CV (< 2.8%) for within-run precision of the test preparation using five instruments (Coagrex-700, Coagrex-800, CA-1500, CA-7000, KC4a micro) produced excellent results (table 2). The result measured by the Jacobsson method based on a manual technique also showed satisfactory precision as well as that of the Clauss method. No significance was observed for between-analyser precision (table 2). Furthermore, no discrepancy in the fibrinogen concentration among instruments with light scattering detection (Coagrex and CA series), mechanical detection (KC-4a) and the manual method (Jacobson) was demonstrated. A homogeneous reaction between the fibrinogen substance and thrombin might have contributed to good precision in the fibrinogen material when using purified fibrinogen with a clear appearance and no contaminants. It has also been reported that plasma material consists of plasma proteins (antithrombin, plasminogen, antiplasmin, coagulation factors) and lipid contaminants (e.g. cholesterol, triglyceride, low- and high-density lipoproteins) [9]. These results suggest that the test preparation with a high purity affects the excellent precision for fibrinogen measurement.

The degradation study was conducted to predict storage time using stressed temperatures. When storage temperature is elevated, a potency of fibrinogen by the Clauss method was gradually decreased. Storage of the test preparation at 45°C for two months or at 37°C for six months resulted in the lyophilized material having good reconstitutability and it gave sufficiently clear clots on the addition of thrombin (data not shown). No effect was observed following storage at 20°C and at lower temperatures (−20 and 4°C). Since it is predicted that the maximum transport time during Japanese distribution would be 1–2 weeks, the maximum transport temperature would be at ambient temperature (up to 30°C) (table 6). The degradation data obtained thus far indicate that the test preparation is a reliable and stable material. It is proposed that a storage temperature is routinely kept at < 4°C for three years or at 9°C for two years. Additional storage studies, however, are currently being undertaken.

In conclusion, the present authors have developed a fibrinogen standard for supply to Japanese medical laboratories and manufacturers that displays high precision and good transport stability using coagulation analysers and it is hoped that the use of this standard as a candidate material for plasma fibrinogen measurement in medical laboratories will help to improve the high rate of inter- or between-laboratory variation observed in Japanese surveillance on various automated or semi-automated coagulation analysers.

### Acknowledgement

The authors are grateful for the technical assistance of Yoshiko Nagao, MT, and Kazuyo Yoshida (International Reagents Corporation, Kobe, Japan) who performed the coagulation analyser assays.

### References

1. Nieuwenhuizen, W., *Euro. Heart J.*, **16** (1995), 6.
2. Gaffney, P. J. and Wong, M., *Thromb. Haemost.*, **68** (1992), 428.
3. Hirai, N., Tatsumi, N., Hino, M., Yamane, T. and Ohta, K., *Osaka City Med. J.*, **44** (1988), 55.
4. Tatsumi, N., Okuda, M., Kondo, H. and Takubo, T., *Proc. Asian Quality Assurance Survey*, 2003 (in press).
5. Tatsumi, N., *Rinsyo Byori*, **49** (2001), 1273.
6. Tatsumi, N., *Rinsyo Byori*, **49** (2001), 1280.
7. Okuda, M., Uemura, Y. and Tatsumi, N., *Prep. Biochem. Biotecnol.*, **33** (2003), 239.
8. Okuda, M., Uemura, Y., Naka, K. and Tatsumi, N., *Clin. Lab. Hematol.*, **25** (2003), 167.
9. Okuda, M., Uemura, Y. and Tatsumi, N., *J. Auto. Met. Manage. Chem.*, **25** (2003), 79.
10. Cohn, F. J., Strong, L. E., Hughes, W. L. Jr., Mulfird, D. J., Ashworth, J. N., Melin, M. and Taylor, H. L., *J. Am. Chem. Soc.*, **68** (1946), 459.
11. Uemura, Y., Yang, Y. H., Heldebrant, C. M., Takechi, K. and Yokoyama, K., *Vox Sang.*, **67** (1994), 337.
12. Whitton, C. M., Sands, D., Hubbard, A. R. and Gaffney, P. J., *Thromb. Haemost.*, **84** (2000), 258.
13. Clauss, V. A., *Acta Haematol.*, **17** (1957), 237.
14. National Committee for Clinical Laboratory Standardization (NCCLS). *Procedure for the Determination of Fibrinogen in Plasma; Approved Guideline*. NCCLS H30-A2 (Wayne: NCCLS, 2001).
15. Kikukawa, N., Okuda, M., Minami, S., Ohta, Y. and Uemura, Y., *Clin. Chem.*, **47** (2001), A167.
16. Okuda, M., Kikukawa, N., Fujikawa, Y., Uemura, Y. and Yokoyama, K., *Clin. Chemistry*, **46** (2000), A135.
17. Jacobsson, K., *Scan. J. Clin. Lab. Invest.*, **7** (1955), 1.
18. Kirkwood, T. B. L., *J. Biol. Stand.*, **12** (1984), 215.

